# Assessment of yield performances for grain sorghum varieties by AMMI and GGE biplot analyses

**DOI:** 10.3389/fpls.2023.1261323

**Published:** 2023-10-30

**Authors:** Runfeng Wang, Hailian Wang, Shaoming Huang, Yingxing Zhao, Erying Chen, Feifei Li, Ling Qin, Yanbing Yang, Yan’an Guan, Bin Liu, Huawen Zhang

**Affiliations:** ^1^ Crop Research Institute, Shandong Academy of Agricultural Sciences, Jinan, Shandong, China; ^2^ Shandong Provincial Engineering Research Center for Featured Minor Crops, Crop Research Institute, Shandong Academy of Agricultural Sciences, Jinan, Shandong, China; ^3^ Crop Development Center, University of Saskatchewan, Saskatoon, SK, Canada

**Keywords:** yield performance and stability, G × E interaction, AMMI, GGE biplot, grain sorghum, multi-environment trial

## Abstract

Grain sorghum is an exceptional source of dietary nutrition with outstanding economic values. Breeding of grain sorghum can be slowed down by the occurrence of genotype × environment interactions (GEI) causing biased estimation of yield performance in multi-environments and therefore complicates direct phenotypic selection of superior genotypes. Multi-environment trials by randomized complete block design with three replications were performed on 13 newly developed grain sorghum varieties at seven test locations across China for two years. Additive main effects and multiplicative interaction (AMMI) and genotype + genotype × environment (GGE) biplot models were adopted to uncover GEI patterns and effectively identify high-yielding genotypes with stable performance across environments. Yield (YLD), plant height (PH), days to maturity (DTM), thousand seed weight (TSW), and panicle length (PL) were measured. Statistical analysis showed that target traits were influenced by significant GEI effects (*p* < 0.001), that broad-sense heritability estimates for these traits varied from 0.40 to 0.94 within the medium to high range, that AMMI and GGE biplot models captured more than 66.3% of total variance suggesting sufficient applicability of both analytic models, and that two genotypes, G3 (Liaoza No.52) and G10 (Jinza 110), were identified as the superior varieties while one genotype, G11 (Jinza 111), was the locally adapted variety. G3 was the most stable variety with highest yielding potential and G10 was second to G3 in average yield and stability whereas G11 had best adaptation only in one test location. We recommend G3 and G10 for the production in Shenyang, Chaoyang, Jinzhou, Jinzhong, Yulin, and Pingliang, while G11 for Yili.

## 1 Introduction

*Sorghum bicolor* L. has a cultivation history of around 5000 years and has been widely planted across the world (da Silva et al., 2021). It has been recognized as the fifth largest cereal crop in the world, following maize, wheat, rice, and barley, with a mean annual production of more than 5.89 million tons over the past five years (FAOSTAT, 2021). Sorghum is highly diverse in its cultivated varieties, each variety owning miscellaneous characteristics, which delivers sorghum versatile utilizations. Therefore, sorghum is considered as an excellent source of human food, animal feed, and bioenergy (Cardoso et al., 2017; Appiah-Nkansah et al., 2019; Dykes, 2019). Grain sorghum is one of the cultivated varieties of the sorghum genus which is primarily used for its grain as human food and animal feed due to the salutary effects of bioactive compounds in the grain (Yun et al., 2019). In China, grain sorghum used to be a staple food source for people. However, it has been replaced by other cereal crops e.g., wheat, maize, and rice with better palatability, and it has become a minor food crop since 1980s (Li et al., 2021). The major utilization purpose of grain sorghum is to produce traditional Chinese liquors with only a small proportion of grain sorghum production consumed as food. Nutritional values of grain sorghum for health benefits have been neglected. Nonetheless, public awareness of healthy living on a diet of miscellaneous grains has spread over the years, which brings opportunities to the utilization of grain sorghum as the source of healthy food and furthermore to the breeding of grain sorghum varieties with excellent traits.

In China, grain sorghum cultivation has been long established and extended across the country. Major grain sorghum production areas cover regions of nine provinces across Northeastern, Northern, Southwestern China (Li et al., 2021). Because of the vast geographical span of the production areas, edaphoclimatic circumstances vary greatly from place to place. The heterogeneity of environments will lead to the differential phenotypic expressions of traits in crops such as sorghum which is defined by the presence of genotypes × environment interactions (GEI) (Yan and Kang, 2003). The occurrence of GEI has the bottleneck effects on grain sorghum improvement breeding because it brings about snags in screening for superior genotypes with high yields and stability as well as genotype recommendation for specific agro-climatic zones (da Silva et al., 2021). Therefore, to neutralize the negative influence of GEI on the outcomes of breeding programs, extensive knowledge on GEI as well as identifying the magnitudes of GEI is supposed to be gained by breeders and researchers in such programs. In recent years, various statistical tools have been developed and aided breeders and researchers in beating the challenges posed by the GEI effects when making decisions on selecting outstanding genotypes for commercial cultivation or promoting genotypes in certain agro-climatic zones.

Analysis of variance (ANOVA) is a commonly used statistical method for multiple mean comparisons across different groups and to extract patterns and trends within complex and varied data. By applying ANOVA, magnitudes of GEI effects on genotypic performances can be drawn. However, the method provides inadequate facts regarding partitioning of significant GEI effects as well as presenting details of how genotypes respond to the effects (Khan et al., 2021). Thus, robust methods and approaches such as the additive main effects and multiplicative interaction (AMMI) (Gauch, 2013) and the genotype + genotype × environment (GGE) biplot (Yan et al., 2000) models that precede ANOVA have been put forward. Both models depend on the principal component analysis (PCA) to decompose MET data and uncover GEI patterns. The AMMI model in combination with ANOVA outputs AMMI1 and AMMI2 biplots, with the first displaying the genotypic means and their relationship to the first PCA and the second showing genotypes’ relationships to the first two PCAs (Bocianowski et al., 2019). Alternatively, GGE biplot analysis incorporates a series of functionalities that assess genotype effects and GEI effects simultaneously to produce “representativeness and discriminativeness” view for test environments, and “which-won-where” pattern, “stability vs. mean performance” as well as “ranking genotypes” views for genotypes in METs (Angelini et al., 2019). Although GGE biplot analysis provides more sophisticated insights into understanding GEI patterns, the two statistical tools complement each other enabling us to comprehend GEI effects (Esan et al., 2023). Aided by these statistical tools, breeders become able to describe how genotypes interact with environments to impact crop output and effectively select superior genotypes (Yan and Kang, 2003). In the present research, METs were carried out on a group of newly developed grain sorghum varieties for the evaluation of yield performance. Data obtained from the METs were subjected to both AMMI and GGE biplot analyses to evaluate GEI effects and identify high-yielding grain sorghum varieties associated with stable adaptability to an extensive range of edaphoclimatic conditions.

## 2 Materials and methods

### 2.1 Plant materials

Plant materials under assessment in the present study were grain sorghum varieties specific for use as human food. Therefore, the seed coat of this type of grain sorghum varieties must contain tannin content less than 0.5%. By the criterion of tannin content, 13 grain sorghum varieties developed by three agricultural sciences research institutions for the spring sowing late maturing regions were collected for evaluation (**Table 1**).

**Table 1:** Specifics concerning 13 grain sorghum varieties under evaluation.

Code	Variety	Tannin (%)	Source	Source location
G1	Liaoza No.10	0.18	Liaoning Academy of Agricultural sciences	Shenyang, Liaoning Province
G2	Liaoza No.48	0.22		
G3	Liaoza No.52	0.23		
G4	Liaoza No.69	0.17		
G5	Liaoza No.72	0.24		
G6	Liaoza No.73	0.19		
G7	Liaonuo No.12	0.26		
G8	Liaonuo No.13	0.18		
G9	Jinza 107	0.18	Jinzhou Academy of Agricultural Sciences	Jinzhou, Liaoning Province
G10	Jinza 110	0.18		
G11	Jinza 111	0.22		
G12	Jinsi 20-1	0.25	Shanxi Agricultural University	Taiyuan, Shanxi Province
G13	Shenza No.5 (Check)	0.19	Liaoning Academy of Agricultural sciences	Shenyang, Liaoning Province

### 2.2 Field trial locations

Since the varieties under evaluation best fitted in the agronomic scenario of spring sowing and late maturing, the field trial locations were accordingly arranged in such regions stretching across seven varying environments located in five administrative regions of China (**Table 2**). Evaluation of their yield performances as well as agronomic traits was carried out by using multi-environment trials at seven test locations in the 2020 and the 2021 growing seasons. Meteorological conditions of the two growing seasons and soil properties of the seven locations were organized in **Supplementary 1**, **2**, respectively.

**Table 2:** Test locations of grain sorghum varieties.

Code	Growing season	Location	Geographical coordinates	Altitude
E1 E8	2020 2021	Shenyang, Liaoning Province	41.80°N, 123.38°E	41.60 m
E2 E9	2020 2021	Chaoyang, Liaoning Province	41.57°N, 120.45°E	168.70 m
E3 E10	2020 2021	Jinzhou, Liaoning Province	41.10°N, 121.13°E	65.90 m
E4 E11	2020 2021	Jinzhong, Shanxi Province	37.68°N, 112.75°E	892.00 m
E5 E12	2020 2021	Yulin, Shaanxi Province	38.29°N, 109.74°E	1057.50 m
E6 E13	2020 2021	Pingliang, Gansu Province	35.55°N, 106.67°E	1346.60 m
E7 E14	2020 2021	Yili, Xinjiang Uygur Autonomous Region	30.75°N, 105.97°E	1231.00 m

### 2.3 Trait measurements

In the growing seasons of 2020 and 2021, yield (YLD) and yield related agronomic traits such as days to maturity (DTM), plant height (PH), panicle length (PL) and thousand seed weight (TSW) were measured for the 13 grain sorghum varieties. The measurements of the traits were repeated three times. A quadrat sampling method was used. Quadrats were randomly chosen for each variety. Each quadrat consisted of six 5-m-long rows of sorghum plants and had an area of 18 m^2^. All panicles within the quadrats were collected for PL and PH measurements, and then threshed for TSW measurement and subsequently converted to YLD.

### 2.4 Field trial management

Common compound fertilizers (15% N, 15% P, 15% K) were applied prior to seeding. The 13 grain sorghum varieties were sown no later than late-May at all test sites both in 2020 and in 2021 growing seasons. Seeding density was restricted at 120,000 plants per ha for each variety according to their growth habits. At the field test sites, each of the 13 grain sorghum varieties was seeded in rows with a spacing of 60 cm.

### 2.5 Statistical analysis

#### 2.5.1 Joint analysis of variance

Joint ANOVA was adopted to detect significant differences Three factors such as genotype, environment, and block within environments are involved in the present study, and then the model used for ANOVA is described below,


[1] $$R_{ijr}\;=m+\;G_{i}+\;E_{j}+\;B_{r}\left(E_{j}\right)+GE_{ij}+\;e_{ijr}$$

where $R_{ijr}$ represents trait response of *i*-th genotype in the *j*-th environment and the *r*-th block, *m* stands for the grand mean, $G_{i}$ is the *i*-th genotype, $E_{j}$ is the emblem of the *j*-th environment, $B_{r}\left(E_{j}\right)$ symbolizes the *r*-th block nested in the *j*-th environment, $GE_{ij}$ represents the interaction effect of the *i*-th genotype and the *j*-th environment, and $e_{ijr}$ is the random error of the *i*-th genotype in the *j*-th environment and the *r*-th block.

#### 2.5.2 Broad-sense heritability

Broad-sense heritability (H^2^) for each trait was estimated by using variance components according to the method reported by (Wang et al., 2019). Since only genotype and environment are involved in the present study, the formula is modified as below,


[2] $$H^{2}=\frac{\delta_{g}^{2}}{\delta_{g}^{2}+\frac{\delta_{gE}^{2}}{E}+\;\frac{\delta_{e}^{2}}{RE}}$$

where $\delta_{g}^{2}$ , $\delta_{gE}^{2}$ , and $\delta_{e}^{2}$ are denoted as variances caused by genotype, genotype × environment interaction, and experimental error, respectively, while *E* and *R* are numbers of environments, and blocks, respectively.

#### 2.5.3 Correlation analysis

Pearson correlation coefficients between two traits were calculated to figure out the inherent associations among the traits under evaluation. The formula used for the calculation is shown below,


[3] $$\rho =\;\frac{\sum_{i=1}^{n}\left(x_{i}-\;\bar{x}\right)\;\times \;\left(y_{i}-\;\bar{y}\right)}{\left(n-1\right)\;\times \;S_{x\;}\times \;S_{y}}$$

where ρ stands for correlation coefficient, $x_{i}$ and $y_{i}$ represent the *i*-th observations for trait x and trait y , $\bar{x}$ and $\bar{y}$ are denoted as the means of trait x and trait y , *n* is the number of rows with no missing data of the trait pair, whereas $S_{x}$ and $S_{y}$ represent the standard deviation for trait x and trait y .

#### 2.5.4 Additive main effects and multiplicative interaction analysis

Mean performance and stability of yield were assessed for the 13 grain sorghum varieties with the computation of AMMI whose model equation is given as follows,


[4] $$y_{ij}=\;\mu \;+\;\alpha_{i}+\;\tau_{j}+\;\sum_{k=1}^{p}\lambda_{k}a_{ik}t_{jk}+\;\rho_{ij}+\;\epsilon_{ij}$$

where, $y_{ij}$ stands for yield response of the *i*-th genotype in the *j*-th environment; μ is the grand mean; $\alpha_{i}$ and $\tau_{j}$ represents the *i*-th genotypic effect and the *j*-th environment effect, respectively, while $\sum_{k=1}^{p}\lambda_{k}a_{ik}t_{jk}+\;\rho_{ij}+\;\epsilon_{ij}$ models the multiplicative genotype × environment interaction effect in which $\lambda_{k}$ stands for the singular value for *k*-th interaction principal component axis (IPCA), $a_{ik}$ is defined as the *i*-th genotype eigenvector for axis *k*, $t_{jk}$ is the *j*-th environment eigenvector for axis *k*, $\rho_{ij}$ stands for the residual not explained by the IPCAs used in the model, whereas $\epsilon_{ij}$ is seen as the error relevant to the model (Olivoto et al., 2019).

#### 2.5.5 AMMI stability indexes

To estimate the 13 grain sorghum genotypes based on stability of their yield performance, two AMMI stability indicators, AMMI stability value (ASV) and weighted average of weighted average of absolute scores from the singular value decomposition of the matrix of best linear unbiased predictions for the genotype × environment interaction effects generated by a linear mixed-effect model and response variable (WAASBY), were computed (Olivoto et al., 2019). For the computation of WAASBY, the 50/50 weight was assigned indicating yield performance and yield stability were attached equal importance in the present study. The formulae shown as follows were used for the calculation of ASV and WAASBY,

for $ASV_{i}$ , AMMI stability value for the *i*-th genotype,


[5] $$ASV_{i}\;=\;\sqrt{\frac{SS_{IPCA1}}{SS_{IPCA2}}{\left(IPCA1\right)}^{2}+\;{\left(IPCA2\right)}^{2}}$$

where IPCA1 and IPCA2 are the scores of IPCA1 and IPCA2 derived from the singular value decomposition (SVD) of the AMMI analysis model, whereas $SS_{IPCA1}$ and $SS_{IPCA2}$ stand for the sum of squares of IPCA1 and IPCA2 scores, respectively (Purchase et al., 2000); to estimate such a superior index that allows weighing between yield performance and stability, WAASBY, the superior index WAASB (weighted average of absolute scores from the singular value decomposition of the matrix of best linear unbiased predictions for the genotype × environment interaction effects generated by a linear mixed-effect model and response variable) needs to be calculated first according to the equation below,


[6] $$WAASB_{i}=\sum_{k=1}^{p}\left|IPCA_{ik}\;\times \;EP_{k}\right|/\sum_{k=1}^{p}EP_{k}$$

where $IPCA_{ik}$ is the score of the *i*-th genotype (or genotype) in the *k*-th IPCA, $EP_{k}$ represents the proportion of variance accounted for by the *k*-th IPCA (Olivoto et al., 2019). To compute WAASBY, YLD (the target trait in the present study) and WAASB index need to be rescaled to the scale of 0 to 100 by using the following equations,


[7] $$rYLD_{i}=\;\frac{100\;-\;0}{YLD_{max}-\;YLD_{min}}\;\times \left(YLD_{i}-\;YLD_{max}\right)+100$$

and


[8] $$rW_{i}=\;\frac{0-100}{W_{max}-\;W_{min}}\;\times \left(W_{i}-\;W_{max}\right)\;+\;0$$

where $rYLD_{i}$ and $rW_{i}$ the rescaled YLD and WAASB values of the *i*-th genotype, respectively, and $YLD_{i}$ and $W_{i}$ are YLD and WAASB values of the *i*-th genotype. Accordingly, the superiority index for the *i*-th genotype that weights between performance and stability, $WAASBY_{i}$ , can be calculated as per the equation below,


[9] $$WAASBY_{i}=\;\frac{\left(rYLD_{i}\;\times \;\theta_{YLD}\right)+\left(rW_{i}\;\times \;\theta_{S}\right)}{\theta_{YLD}+\;\theta_{S}}$$

where $\theta_{YLD}$ and $\theta_{S}$ stand for the weights assigned to YLD and stability (Olivoto et al., 2019).

#### 2.5.6 GGE biplot analysis

GGE biplot analysis is implemented when there are significant effects of genotype × environment interactions confusing the straightforward phenotypic screening of superior genotypes. The most common model used for this biplot analysis is with the singular value decomposition centralized on either genotype or environment. This can be done with the formula as follows,


[10] $${\hat{Y}}_{ij}=\;\mu +\;\beta_{j}+\;\lambda_{1}\xi_{i1}\eta_{1j}+\;\lambda_{2}\xi_{i2}\eta_{2j}+\;\epsilon_{ij}$$

where ${\hat{Y}}_{ij}$ is defined as the expected yield of the *i*-th genotype in the *j*-th environment, μ is the grand yield mean, $\beta_{j}$ is the main effect of the *j*-th environment, $\lambda_{1}$ and $\lambda_{2}$ are the singular values of the first two principal components, PC1 and PC2, respectively, $\xi_{i1}$ and $\xi_{i2}$ are the eigenvectors of the *i*-th genotype for PC1 and PC2, respectively, whereas $\eta_{1j}$ and $\eta_{2j}$ are the eigenvectors of the *j*-th environment for PC1 and PC2, respectively, $\epsilon_{ij}$ is the residual that cannot be explained by G or GE effect (Yan and Kang, 2003).

### 2.6 Computation and visualization for trait correlations and biplot analyses

Correlation studies of agronomic traits, mean performances and stability assessments of yield by AMMI and GGE biplot analyses were conducted and visually presented with the package “metan” ver. 1.8.0 in R ver. 4.2.2 (Olivoto and Lucio, 2020). The following models were used to output biplots for visualization of the GEI effects on yields: AMMI1 and AMMI2 of AMMI, and “relation among environments”, “discriminativeness vs. representativeness”, “mean vs. stability”, “ranking genotypes”, and “which-won-where” views of GGE biplot.R scripts for trait correlations, AMMI, and GGE biplot visualization are presented in **Supplementary 3**–**5**, respectively.

## 3 Results

### 3.1 Natural variation, correlation, and mean performance of yield and agronomic traits contributing to yield

**Figure 1:**
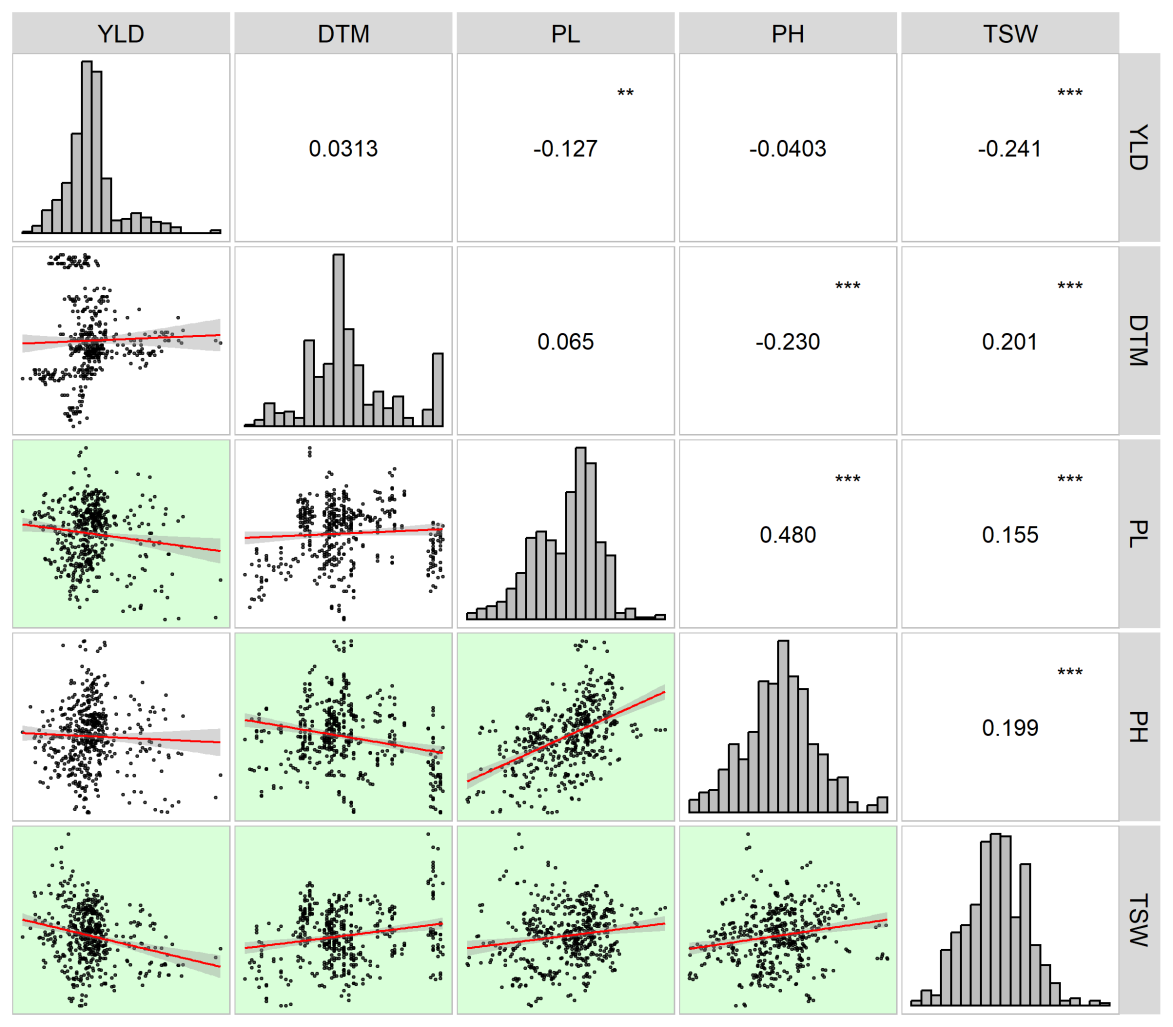
Correlogram of yield (YLD) and yield contributing traits (DTM, days to maturity; PL, panicle length; PH, plant height; TSW, thousand seed weight). Histograms are placed along the diagonal line with coefficients of correlations between the traits in the upper right corner while scatterplots with regression lines corresponding to the correlations between the traits in the lower left corner. Significant correlations are highlighted with the green background in the scatterplots. ** stands for the significance level at *p*< 0.01 whereas *** represents the significance level at *p*< 0.001.

Field trials of two growing seasons revealed large natural variation for both yields and agronomic traits that contributed to the overall yields of the 13 sorghum varieties. Raw data obtained from the field trails are available in **Supplementary 6**. As shown in **Table 3**, yields of the 13 sorghum varieties ranged from 1.08 t·ha^-1^ to 22.32 t·ha^-1^ and were averaged at 8.04 t·ha^-1^. In terms of average yield, G3 had the highest value of 9.33 t·ha^-1^ while G7 had the smallest value of 7.23 t·ha^-1^. The average DTM was 126.11 d and it ranged from 84.00 d to 166.00 d. G13 was the earliest mature variety whereas G8 took the longest days, 128.14 d, to fully mature. G1 was featured by the longest average PL of 32.91 cm while the shortest PL was observed for G2 with the length being 28.11 cm. Across all the growing environments, PL ranged from 19.25 cm to 43.56 cm. The performance of PH varied largely with environments. PH ranged from 98.64 cm to 251.49 cm. G5 had the highest mean value of 192.55 cm for PH, while G4 was the shortest variety with a PH value of 137.78 cm. Variation for TSW was in between 18.36 g and 45.53 g with a mean of 29.35 g for all sorghum varieties. The variety that had the highest TSW was G13, 32.04 g. On the other hand, TSW of G10 was the lowest of all varieties being 27.10 g. Yield and yield contributing traits were interconnected as revealed by the correlation analysis (**Figure 1**). Yield had significant negative correlations between some of those agronomic traits under evaluation. For example, YLD was negatively correlated with PL (r = -0.127, *p*< 0.01) and TSW (r = -0.241, *p*< 0.001), indicating that the grain sorghum genotypes studied in the present study that had higher production had lower panicle length and thousand seed weight. Furthermore, relations between YLD and other traits such as DTM and PH were seemingly weak. On the other hand, between some agronomic traits were seen significant correlations. As shown in **Figure 1**, TSW had significantly positive correlation with DTM (r = 0.201, *p*< 0.001), PL (r = 0.155, *p*< 0.001), and PH (r = 0.199, *p*< 0.001). Additionally, PH was positively correlated with PL (r = 0.480, *p*< 0.001), whereas negatively correlated with DTM (r = -0.230, *p*< 0.001).

**Table 3:** Mean performances of yields and agronomic traits in the 13 sorghum varieties.

Var	YLD (t·ha^-1^)				DTM (d)				PL (cm)				PH (cm)				TSW (g)			
	Mean	SD	CV (%)	Range	Mean	SD	CV (%)	Range	Mean	SD	CV (%)	Range	Mean	SD	CV (%)	Range	Mean	SD	CV (%)	Range
G1	8.19	3.57	43.58	15.94	126.15	15.19	12.04	64.00	32.91	2.91	8.85	10.86	186.83	22.90	12.26	91.78	30.12	4.23	14.05	15.71
G2	7.82	3.07	39.25	14.66	125.29	20.12	16.06	77.00	28.11	1.75	6.21	7.50	151.68	14.97	9.87	63.96	30.12	5.09	16.90	21.88
G3	9.33	2.68	28.66	11.38	127.36	18.51	14.54	67.00	29.52	2.68	9.07	11.18	150.94	16.87	11.18	64.59	29.99	4.45	14.83	15.77
G4	7.53	3.39	45.00	14.82	126.23	18.64	14.77	79.00	31.44	4.26	13.53	13.84	137.78	18.49	13.42	63.66	28.46	4.36	15.33	14.88
G5	7.42	2.56	34.48	12.04	127.79	19.88	15.56	76.00	32.45	3.61	11.12	13.34	192.55	28.93	15.02	113.20	29.84	5.57	18.67	19.06
G6	7.80	2.58	33.08	13.53	126.00	16.80	13.33	65.00	31.49	3.41	10.83	11.70	156.99	19.12	12.18	68.56	28.21	3.70	13.12	14.01
G7	7.23	2.56	35.39	12.52	126.46	18.21	14.40	71.00	31.71	4.06	12.80	14.43	174.39	28.13	16.13	102.96	28.52	3.85	13.49	14.49
G8	7.99	2.76	34.56	13.31	128.14	18.40	14.36	68.00	31.84	2.51	7.89	9.59	161.04	17.47	10.85	72.55	29.51	2.97	10.07	13.47
G9	7.69	2.72	35.39	11.23	124.92	18.81	15.05	76.00	31.76	3.94	12.40	16.96	177.59	30.81	17.35	136.62	29.10	2.22	7.62	8.61
G10	9.24	2.88	31.21	12.49	126.00	16.47	13.07	66.00	30.01	3.12	10.38	11.93	153.57	25.54	16.63	92.11	27.10	4.73	17.46	18.54
G11	8.66	3.90	45.10	18.28	128.07	19.47	15.20	73.00	32.31	5.31	16.43	18.62	175.44	24.50	13.96	101.44	28.40	4.89	17.22	18.28
G12	8.00	3.37	42.12	18.96	123.31	17.94	14.55	75.00	32.78	6.40	19.52	21.55	160.25	13.69	8.54	50.44	30.19	5.26	17.42	22.67
G13	7.58	2.83	37.31	11.48	123.23	17.98	14.59	76.00	31.64	5.11	16.15	16.59	186.48	27.06	14.51	115.15	32.04	4.11	12.81	15.12
MaE	22.32	–	–	–	166.00	–	–	–	43.56	–	–	–	251.49	–	–	–	45.53	–	–	–
MiE	1.08	–	–	–	84.00	–	–	–	19.25	–	–	–	98.64	–	–	–	18.36	–	–	–
MeE	8.04	–	–	–	126.11	–	–	–	31.38	–	–	–	166.58	–	–	–	29.35	–	–	–
SDE	3.05	–	–	–	18.12	–	–	–	4.14	–	–	–	27.77	–	–	–	4.47	–	–	–
CVE (%)	37.98	–	–	–	14.37	–	–	–	13.20	–	–	–	16.67	–	–	–	15.22	–	–	–

Var, variety; YLD, yield; DTM, days to maturity; PL, panicle length; PH, plant height; TSW, thousand seed weight; SD, standard deviation; CV, coefficient of variation; MaE, maximun value across environments; MiE, minimum value across environments; MeE, mean value across environments; SDE, standard deviation across environments; CVE, coefficient of variation across environments; -, not applicable.

### 3.2 Joint analysis of variance for yield and agronomic traits contributing to yield

To analyze the effects of genotype, environment, and their multiplicative interaction on yields and other traits impacting yields, field data of trait performances were subjected to joint ANOVA to estimate mean squares and resultantly calculate broad-sense heritability (complete ANOVA tables for all traits studied are available in **Supplementary 7**). The present study revealed significant variations for YLD, DTM, PL, PH and TSW (**Table 4**). Genotypes had significant effects on YLD (*p*< 0.001), DTM (*p*< 0.001), PL (*p*< 0.001), PH (*p*< 0.001) and TSW (*p*< 0.001). On the other hand, notably significant fluctuation over environments was detected for all traits of interest (*p*< 0.001). An explanation for that could be the uneven climatic conditions of the growing locations as well as the growing seasons that all together altered the agronomic performances of the traits under evaluation in the present study. Likewise, the interaction between genotypes and environments (G × E) gave rise to significantly different mean squares of these traits (*p*< 0.001). Replications nested in environments, i.e., R (E), produced insignificant discrepancies for YLD, DTM, PL, and PH (*p* > 0.05) while mean square caused by TSW was noticed substantially different for R (E) (*p*< 0.05). In terms of broad-sense heritability (H^2^), DTM (0.40) and TSW (0.52) had medium H^2^ estimates (0.30 ~ 0.60) as per scale of H.W. Zhang et al. (2019). However, the traits, PH (0.94), YLD (0.86), and PL (0.71) were highly heritable (> 0.60).

**Table 4:** Sum of squares, mean squares and broad-sense heritability (H^2^) estimation by ANOVA for yield and agronomic traits.

Source	YLD		DTM		PL		PH		TSW	
	SS	MS	SS	MS	SS	MS	SS	MS	SS	MS
G	2.21×10^2^	18.41^***^	1.24×10^3^	1.03×10^2***^	5.60×10^2^	79.98^***^	1.42×10^5^	1.18×10^4***^	7.26×10^2^	60.48^***^
E	3.91×10^3^	3.01×10^2***^	1.64×10^5^	1.26×10^4***^	4.69×10^3^	3.61×10^2***^	1.68×10^5^	1.29×10^4***^	4.96×10^3^	4.13×10^2***^
G × E	4.20×10^2^	2.70^***^	5.74×10^3^	38.79^***^	3.60×10^3^	23.07^***^	1.08×10^5^	6.94×10^2***^	4.34×10^3^	30.12^***^
R (E)	45.60	1.63^ns^	51.00	1.84^ns^	9.40	0.34^ns^	2.34×10^2^	8.35^ns^	10.50	0.41^*^
Err	4.77×10^2^	1.42	4.29×10^2^	1.34	88.54	0.26	2.49×10^3^	7.40	71.20	0.23
H^2^	0.86		0.40		0.71		0.94		0.52	

SS, sum of squares; MS, mean squares; YLD, yield; DTM, days to maturity; PL, panicle length; PH, plant height; TSW, thousand seed weight; G, genotype; E, environment; R, replication; Err, error; H^2^, broad-sense heritability; ^ns^, not significant; ^*^, and ^***^, significant at p-value of 0.05, and 0.001, respectively.

### 3.3 AMMI model analysis for yield

Yield data of the 13 grain sorghum varieties were collected from each test site and each growing season, and were subjected to the AMMI analysis by using the metan package in R. According to the analytic results (**Table 5**), genotype (G), environment (E), and the genotype and environment interaction (G × E) had significant effects on yields (*p*< 0.001) which was verified in the joint ANOVA (**Table 4**). Additionally, for the significant G × E effect, 12 interaction principal component axes (IPCAs) were detected. Only the first two of them had significant differences (*p*< 0.01). The two IPCAs with significant differences had 24 and 22 degrees of freedom and explained 51.5% and 14.8% of the multiplicative interaction effect, respectively, with the cumulative proportion of 66.3% of the G × E interaction (GEI).

**Table 5:** Additive main effects and multiplicative interaction (AMMI) analysis table.

Source	Df	Sum Sq	Mean Sq	Proportion (%)	Accumulated (%)
E	13	3.91×10^3^	3.01×10^2***^	–	–
R (E)	28	45.60	1.63^ns^	–	–
G	12	2.21×10^2^	18.41^***^	–	–
G × E	156	4.20×10^2^	2.70^***^	–	–
PC1	24	2.16×10^2^	9.02^***^	51.5	51.5
PC2	22	62.00	2.82^**^	14.8	66.3
PC3	20	38.00	1.90^ns^	9.0	75.3
PC4	18	34.30	1.91^ns^	8.2	83.4
PC5	16	26.30	1.64^ns^	6.2	89.7
PC6	14	21.40	1.53^ns^	5.1	94.8
PC7	12	7.17	0.60^ns^	1.7	96.5
PC8	10	6.02	0.60^ns^	1.4	97.9
PC9	8	3.76	0.47^ns^	0.9	98.8
PC10	6	2.99	0.50^ns^	0.7	99.5
PC11	4	1.95	0.49^ns^	0.5	100.0
PC12	2	8.81×10^-3^	4.41×10^-3ns^	0.0	100.0
Residuals	336	4.77×10^2^	1.42	–	–
Total	701	5.50×10^3^	7.84	–	–

Df, degree of freedom; Sum Sq, sum of squares; Mean Sq, mean of squares; Pr, probability; E, environment; R, replication; G, genotype; PC, principal component; -, not applicable; ^ns^, not significant; ^**^, and ^***^, significant at p-value of 0.01, and 0.001, respectively.

#### 3.3.1 Mean values, interaction principal component scores and stability indices of yield

Pooled field data showed that the yield means of the 13 sorghum varieties varied between 7.23 t·ha^-1^ and 9.33 t·ha^-1^ in the sequence of G3 > G10 > G11 > G1 > G12 > G8 > G2 > G6 > G9 > G13 > G4 > G5 > G7 (**Table 6**). Stability of the field performance of yield can be measured by the comparison of IPCA scores as well as stability indexes computed by the AMMI model. Genotypes with IPCA1 scores closer to zero indicate lower influence of GEI effect and therefore higher stability (Q. Liu et al., 2022). For that reason, stability, in terms of IPCA scores, of each sorghum variety was G13 > G3 > G9 > G10 > G2 > G4 > G8 > G12 > G1 > G6 > G5 > G7 > G11. On top of IPCA scores, AMMI model analysis also calculated stability indexes (**Table 6**). For ASV, stability of the sorghum varieties was in the order of G3 > G9 > G10 > G2 > G4 > G13 > G8 > G12 > G1 > G6 > G5 > G7 > G11. However, WAASBY indexes had some inconsistencies for the stability order compared with the ASV values of the varieties, i.e., G3 > G10 > G2 > G8 > G9 > G13 > G4 > G12 > G1 > G6 > G11 > G5 > G7. Notably, WAASBY is a superior stability index that ranks genotypes based on both stability and mean performance at various weights for stability and mean performance (Olivoto et al., 2019). In the present study, stability and mean performance are equally important (at 50/50 weight), and therefore, sorghum varieties with both high stability and high mean performance of yield were regarded as the desirable varieties. In such a selection scenario, considering the three stability evaluation results, although G11 was a high yielding variety, its field performance of yield was less stable than G3 and G10 that had both high yields as well as high stability and thus were thought to be the ideal sorghum varieties.

**Table 6:** Mean values, IPCA scores, and stability indexes for yield across all environments.

Var	YLD (t·ha^-1^)	YLD.R	IPCA score		Stability index and rank			
			IPCA1	IPCA2	ASV	ASV.R	WAASBY	WAASBY.R
G1	8.19	4	0.788	1.130	2.970	9	42.507	9
G2	7.82	7	0.320	0.164	1.130	4	59.342	3
G3	9.33	1	0.204	0.109	0.719	1	100.000	1
G4	7.53	11	0.335	0.361	1.220	5	50.041	7
G5	7.42	12	-1.020	-0.652	3.610	11	21.042	12
G6	7.80	8	-0.921	0.428	3.240	10	35.568	10
G7	7.23	13	-1.480	0.432	5.200	12	2.615	13
G8	7.99	6	-0.426	0.304	1.520	7	58.464	4
G9	7.69	9	-0.271	-0.247	0.978	2	57.157	5
G10	9.24	2	0.307	0.155	1.080	3	93.762	2
G11	8.66	3	1.640	-0.144	5.740	13	33.919	11
G12	8.00	5	0.676	-0.936	2.540	8	43.851	8
G13	7.58	10	-0.152	-1.110	1.230	6	50.193	6

Var, variety; YLD, mean value of yield; YLD.R, rank based on YLD; IPCA, interaction principal component axis; ASI, AMMI stability index; ASI.R, rank based on ASI.R; ASV, AMMI-stability value; ASV.R, rank based on ASV; WAASBY, superior index, weighted average of WAASB (weighted average of absolute scores from the singular value decomposition of the matrix of best linear unbiased predictions for the genotype × environment interaction effects generated by a linear mixed-effect model) and response variable; WAASBY.R, rank based on WAASBY.

#### 3.3.2 AMMI1 biplot analysis

**Figure 2:**
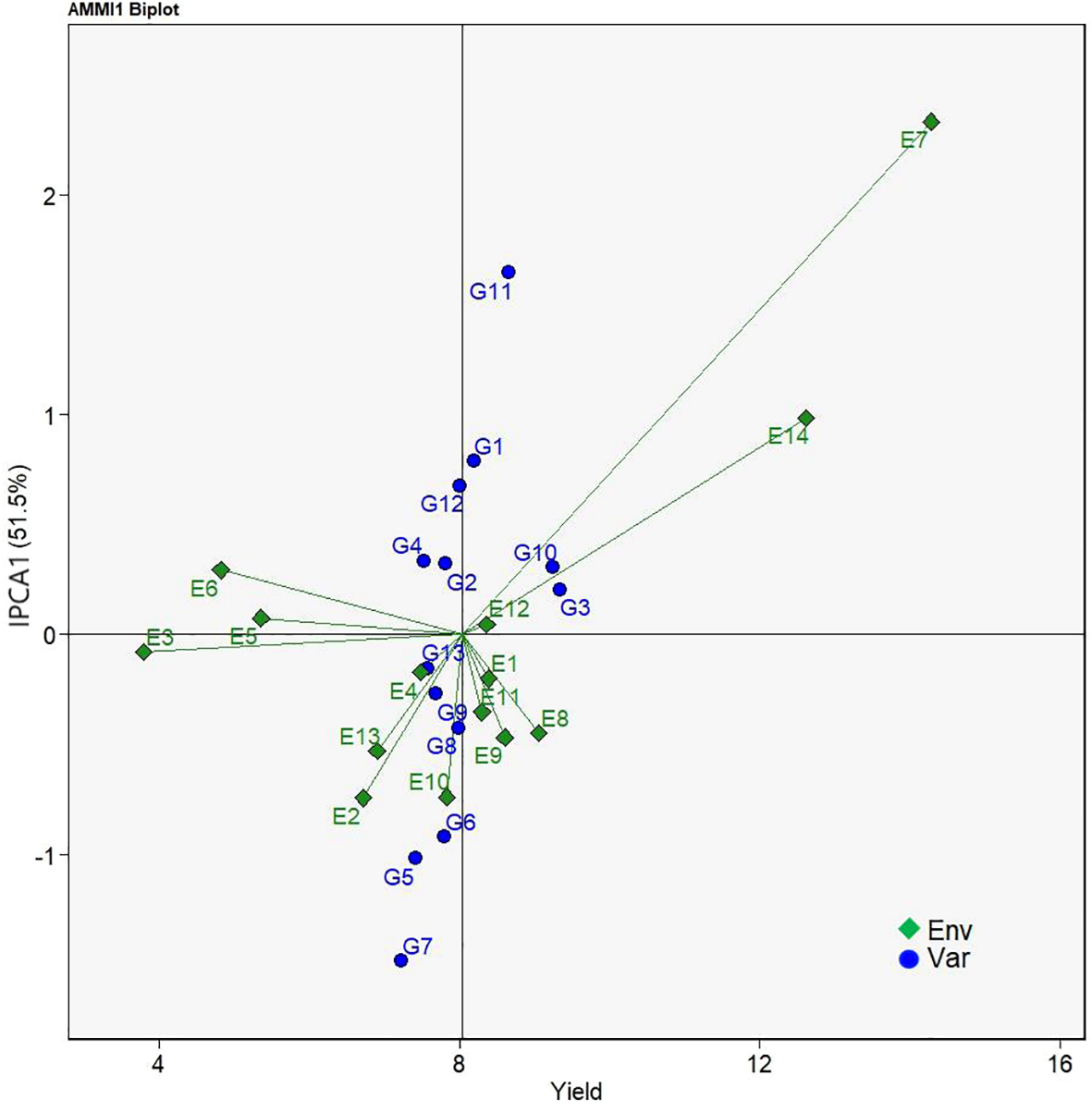
AMMI1 biplot based on IPCA1 (ordinate) and mean yield (abscissa) performances showing genotype × environment interactions of 13 grain sorghum varieties in 14 test environments.

AMMI biplot analysis serves as an informative tool to assess genotypes’ stability over a serial of field test conditions. AMMI1 biplot projects genotypes onto the ordinate and the abscissa representing the additive main effect of genotypes and the effects of interplay between genotype and environment, respectively (Khan et al., 2021). In **Figure 2**, the abscissa represented the yield means of the sorghum varieties while the ordinate stood for the scores of the IPCA1 of each sorghum variety. The origin on both the abscissa and the ordinate axes signified the average yield and zero effects of genotype and environment interaction. Yield means on the right side of abscissa axis were greater than those on the left side. On the other hand, deviations from the origin along the ordinate axis meant environmental influences on yield means. Locations of genotypes farther away from the abscissa axis demonstrated the greater environment effects on yield means, and thus less stability. Consequently, G3, G10, G11, and G1 had yields above the average yield of all sorghum varieties. As for stability, G13, G3, G10, G2, G9 were relatively close to the abscissa on the biplot, indicating the stability of G13 > G3 > G10 > G2 > G9. Considering both yield means and stability, G1, G2, G3, G9, and G10 either had higher stability but lower yield mean performance or higher yield mean performance but low stability. Only the two sorghum varieties, G3 and G10, had higher yields but lower level of interaction with environment and were selected as the best sorghum varieties.

#### 3.3.3 AMMI2 biplot analysis

**Figure 3:**
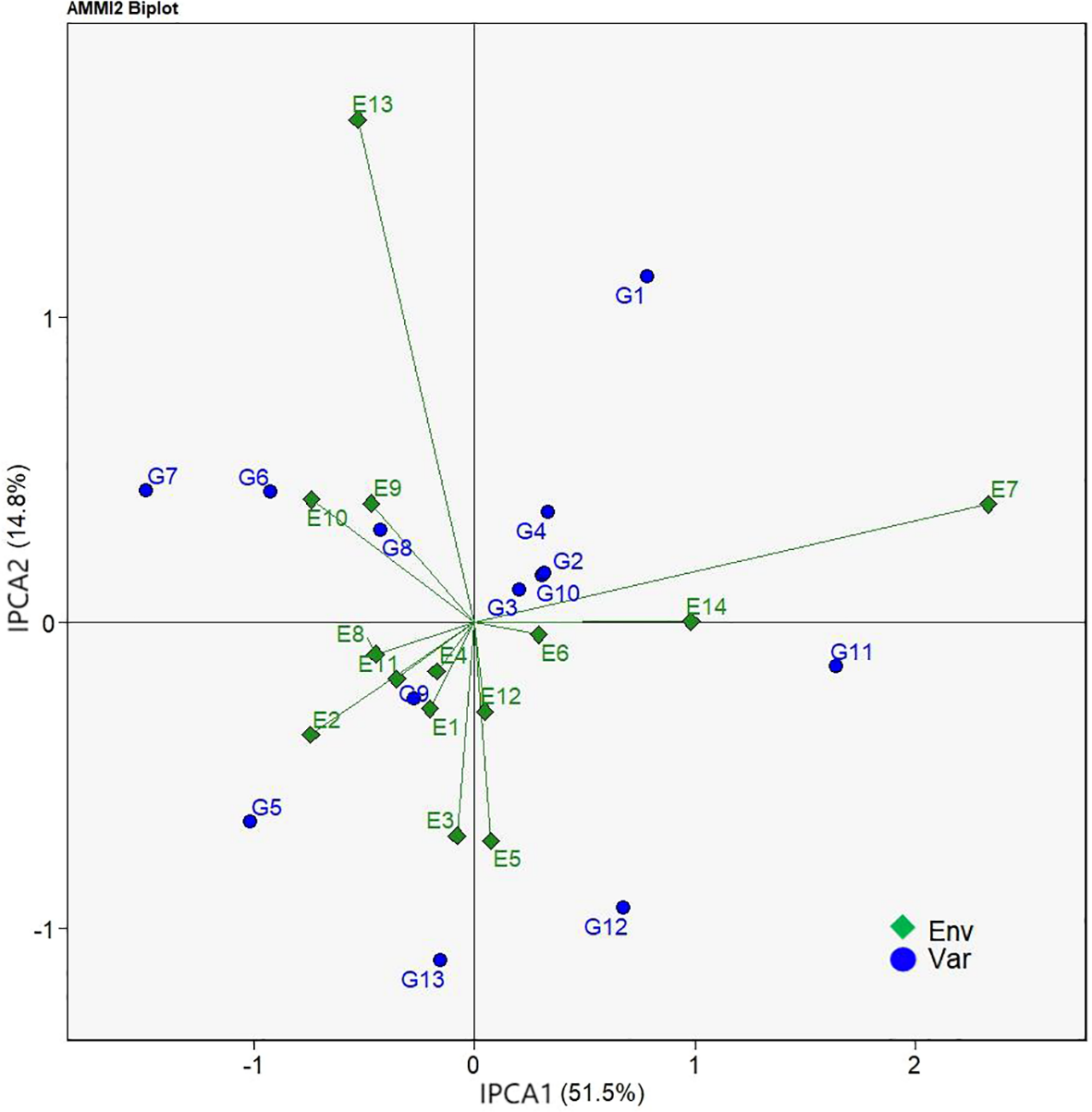
AMMI2 biplot based on IPCA1 (abscissa) and IPCA2 (ordinate) showing genotype × environment interactions of 13 grain sorghum varieties in 14 test environments.

Unlike AMMI1 biplot that infers the impacts of environmental effects on main genotype effect, AMMI2 biplot is an IPCA1- and IPCA2-score based approach to visualize the effects of genotype and environment interaction on genotype ranking (Khan et al., 2021). By this means, genotypes located closer to the origin of the AMMI2 biplot have lower influences of genotype by environment interaction and thus higher stability across environments. **Figure 3** showed that distance of G3 from the biplot origin was the shortest demonstrating that the G3 variety had the strongest adaptability of all test varieties. Following G3, the genotypes G10 and G2 were the genotypes that had high stability. Genotypes such as G1, G7, G5, G13, and G11 that interacted positively or negatively with environments were located farther from the biplot origin indicating their unstable yield performance over different environments.

### 3.4 GGE biplot analysis

The GGE biplot technique was used in the present study as an alternative scheme to provide informative insights into the identification of ideal genotypes in addition to the evaluation of genotypes for mega-environmental delineation.

#### 3.4.1 Relations between environments

**Figure 4:**
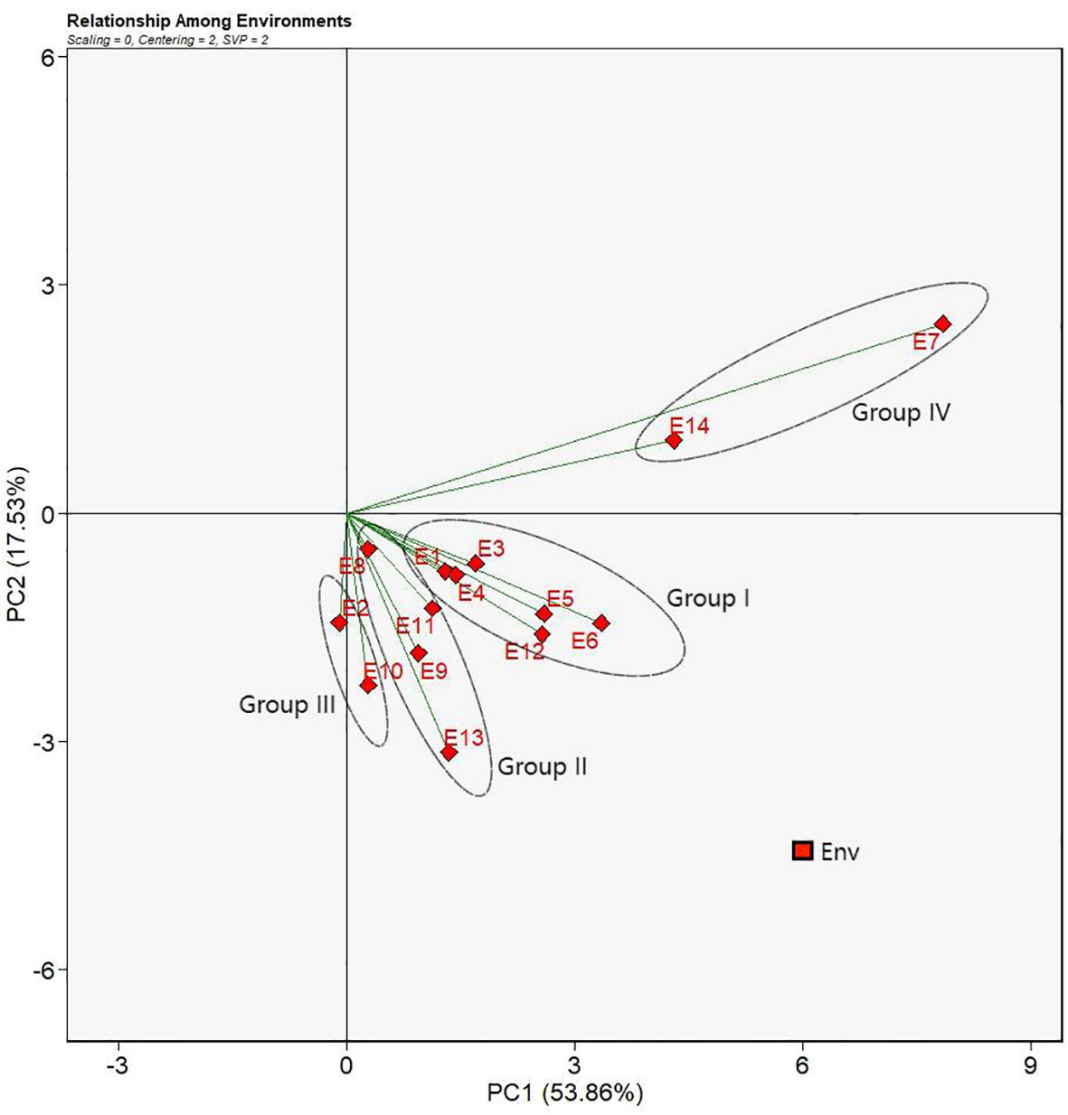
Relation between environments view of the GGE biplot for 14 test environments. The biplot was generated with scaling = 0, centering = 2, and singular value partitioning = “environment”.

Singular value decomposition (SVD) revealed the first two principal components (PCs) that explained 53.86% and 17.53% of the total Genotype + Genotype × Environment (G + G × E) variation. Relations between environments were presented in **Figure 4**. Smaller angle between two environments means higher correlation between the two environments demonstrating co-occurrence for the genotype ranking or high level of repeatability, whereas the larger angle between two environments indicates less correlation or contradicting genotypic performance (Habtegebriel, 2022). Therefore, E1, E3, E4, E5, E6, and E12 had smaller angles between one another forming a group of environments (Group I) where the sorghum genotypes had comparable performance and genotypic ranks. Accordingly, similar patterns were observed for E8, E9, E11, and E13 (Group II), E2 and E10 (Group III), and E7 and E14 (Group IV). However, environments between the group of E7and E14 and another group of E8, E9, and E13 had angles nigh on 90°. It suggested that genotypes under those environments had irrelevant genotypic performances. Moreover, angles between the group of environments, E7 and E14, and another group of E2 and E10 were obtuse which suggested that genotypic performances were dissimilar and even opposite in terms of genotypic ranking based on yields.

#### 3.4.2 Discriminativeness and representativeness of environments

**Figure 5:**
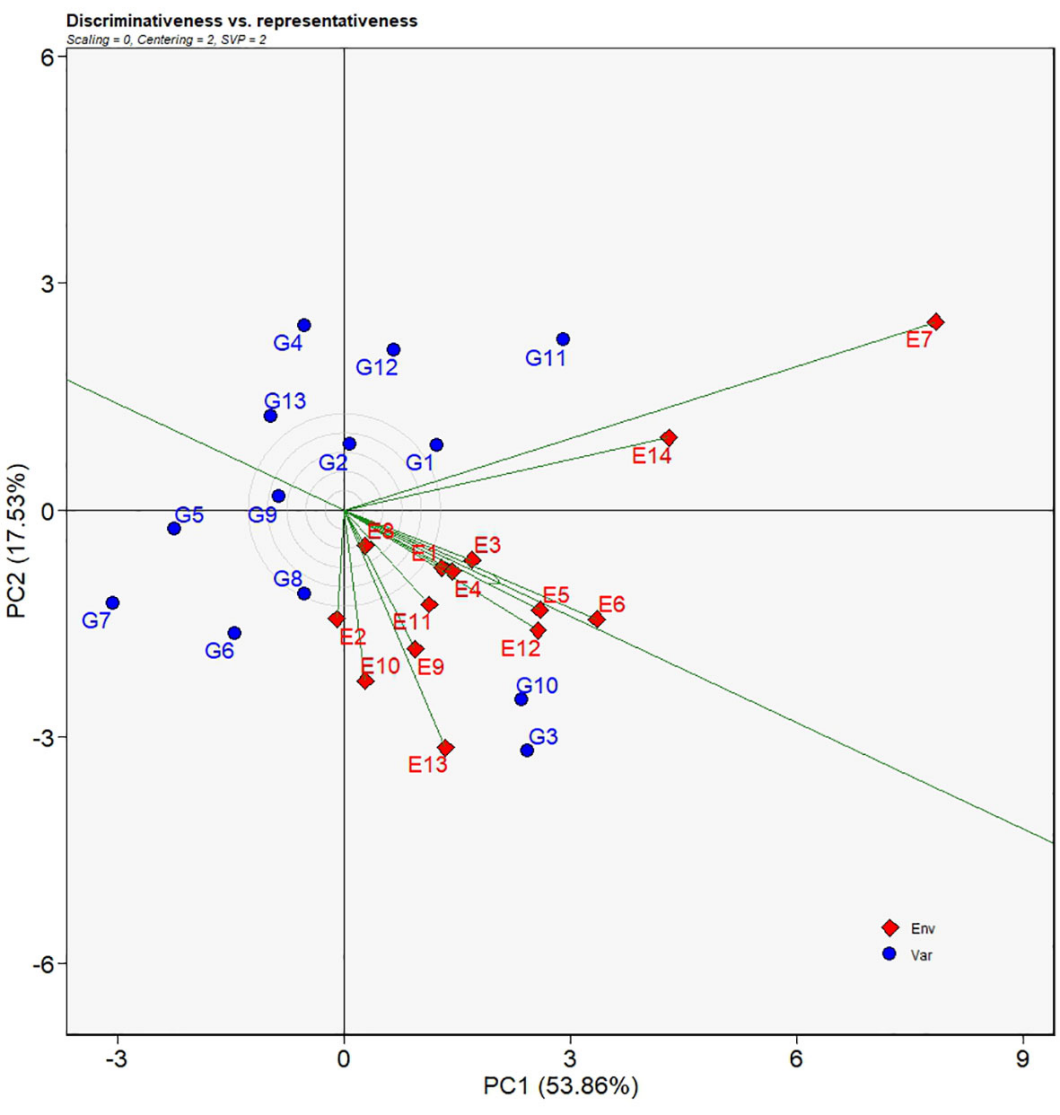
Discriminativeness VS. representativeness view of the GGE biplot for 14 test environments. The biplot was generated with scaling = 0, centering = 2, and singular value partitioning = “environment”.

The discriminating force of each environment can be assessed by the environment vector which is defined as the length of line segment connecting the biplot origin and the environment. Longer length of a vector implies stronger discriminativeness for a given environment. In **Figure 5**, the vector E7 had the longest length and therefore genotypes under such an environment had well distinguished yields. Environments such as E14, E13, E6, E12, E5, E10, and E11 had relatively longer lengths suggesting those environments had a good discriminating force. In contrast, E8 had the shortest vector length from the biplot origin which was thought to be the least distinguishing environment. Genotypes under E8 environment showed similar yields. The line segment with an arrow that crossed the biplot headed downwards at an angle was regarded as the average environment coordinate (AEC) axis (**Figure 5**). In relation to the AEC axis, E5 and E6 had the lowest angle implying that the two environments were the most representative environments. Greater angles relative to the AEC axis meant less representative of environments, for example, E7, E14, E2, E10 and so forth.

#### 3.4.3 Mean performance, stability, and genotype ranking

**Figure 6:**
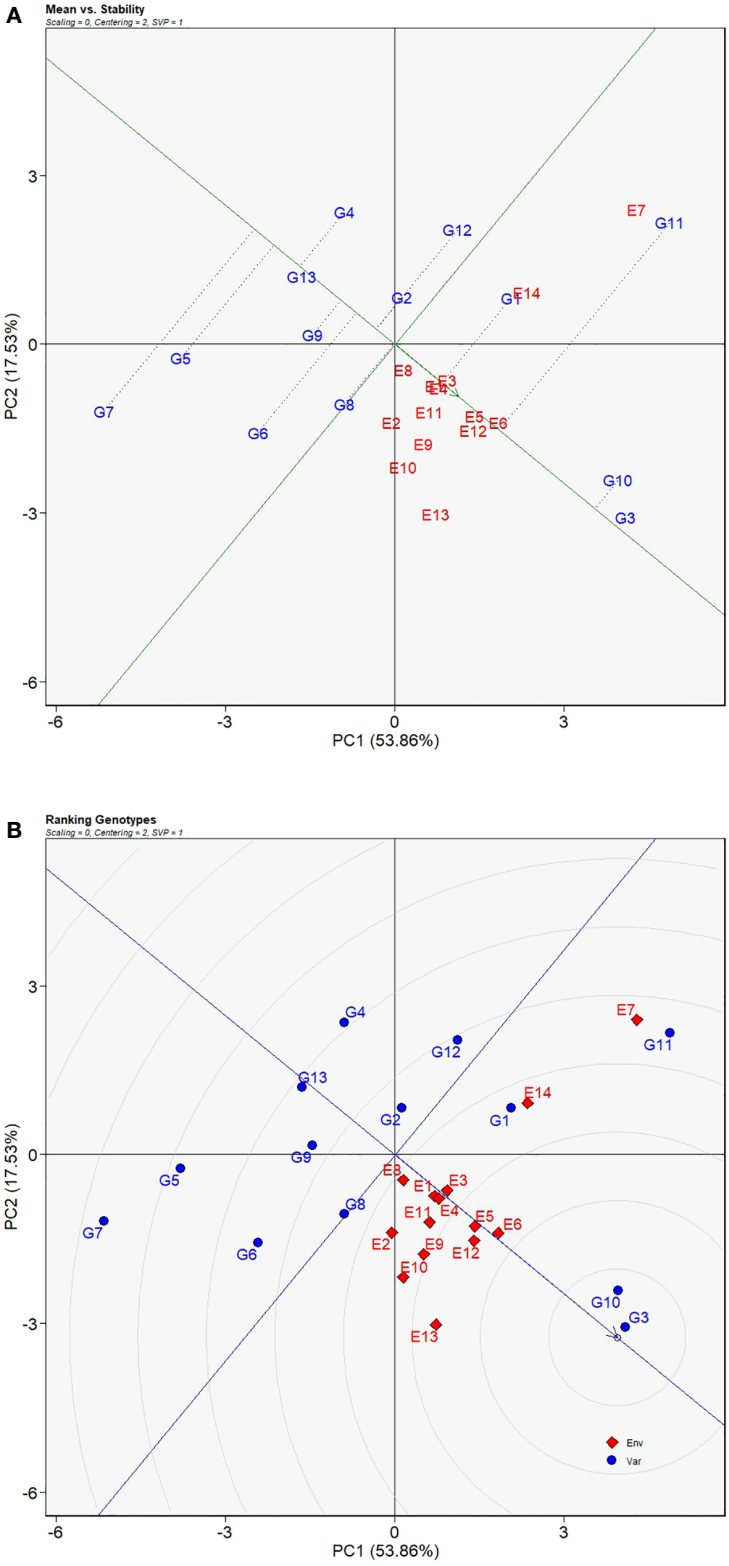
Mean VS. stability view **(A)** and ranking genotype view **(B)** of GGE biplot for average yields of 13 grain sorghum varieties influenced by genotype × environment interactions across 14 test environments. The biplot was generated with scaling = 0, centering = 2, and singular value partitioning = “genotype”.

Genotypes located on the right along the AEC axis have greater yield means and moreover vertical distances, upwards or downwards, indicate magnitudes of GEI effects. As shown in **Figure 6A**, G3 had the highest yield means tightly followed by G10, and then G11 and G1 whose yield means were above the average yield means of all sorghum varieties. With respect to yield performance stability, **Figure 6A** showed that G13 had the least deviation from the AEC axis suggesting its strongest stability across environments which was followed by G3, a genotype adjacent to the AEC axis, G10, G2, and then G9, demonstrating that the four genotypes had less influence of the GEI effects. Although the genotypes mentioned above had excellent stability or yield performance, only G3 and G10 were the steadily productive genotypes across most of the test sites. To be more exact, genotypic ranking was illustrated in the ranking genotype view of the GGE biplot (**Figure 6B**) in which genotypes closer to the center of the concentric circles had better ranks. For that reason, the ranking of these genotypes was in the order of G3 > G10 > G1 > G8 > G11 > G2 > G12 > G9 > G6 > G13 > G4 > G5 > G7.

#### 3.4.4 Which-won-where patterns for yield

**Figure 7:**
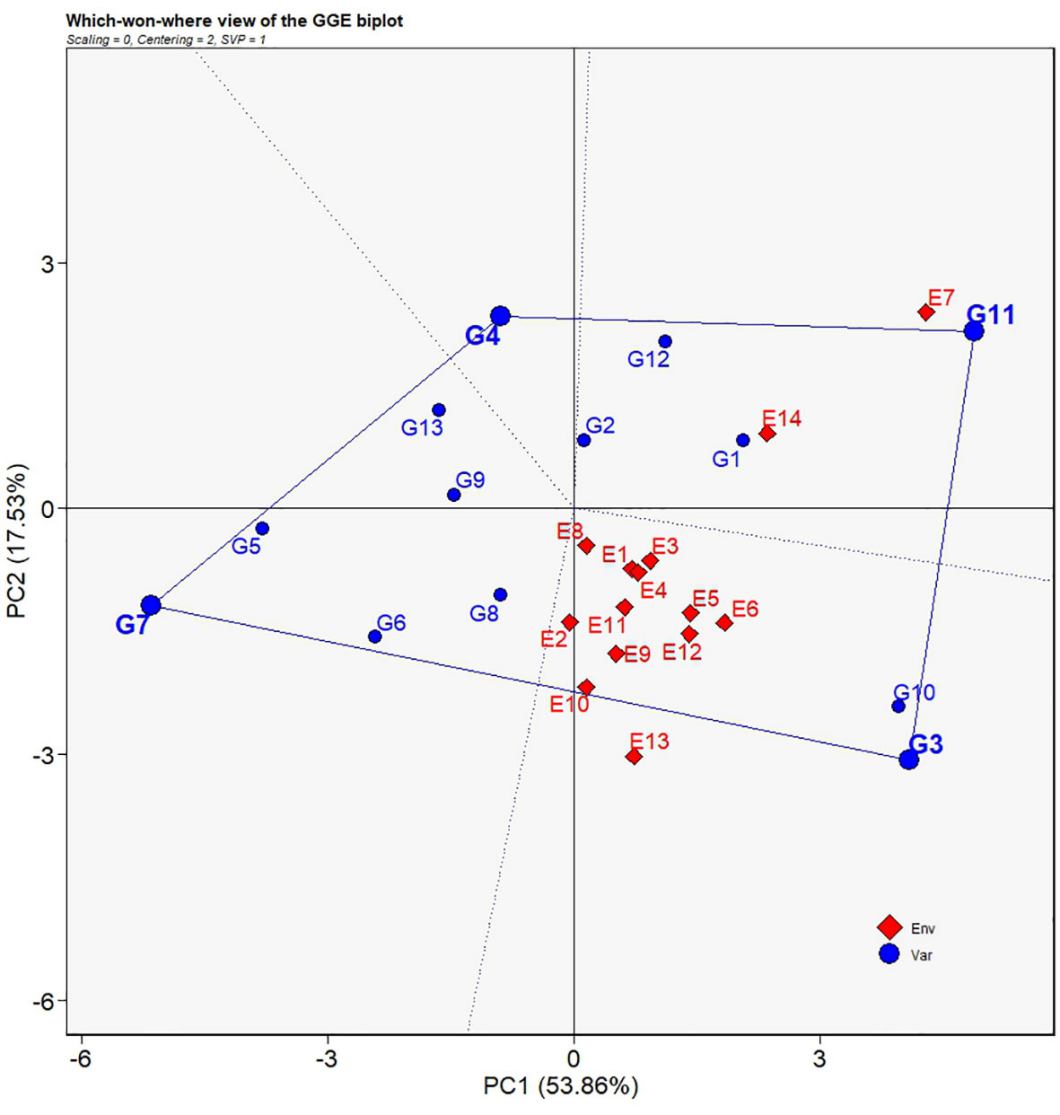
Which-won-where view of the GGE biplot for average yields of 13 grain sorghum varieties in 14 test environments. The biplot was generated with scaling = 0, centering = 2, and singular value partitioning = “genotype”.

The polygon view of the GGE biplot indicates the which-won-where patterns for yield performances of the 13 grain sorghum varieties (**Figure 7**). The dotted lines perpendicular to the borders of the polygon divide the biplot into four regions. Each region containing different environments forms a group of environments. In the present study, E7 and E14 fell into a group of environments while all the other environments formed a second group. Vertexes of the polygon represent the genotypes that have best performance in the corresponding groups of environments. Accordingly, G11 was regarded as the best sorghum variety only in both E7 and E14, whereas G3 was the ideal sorghum variety in all the other environments and had broad adaptability over other varieties.

## 4 Discussion

### 4.1 Yield and yield contributing traits influenced by genotype and environment interaction

Grain sorghum has long been mostly used as an essential flavor-producing material to make liquors in China. Only recently has the use of grain sorghum as human food grown in popularity due to the nutritional facts that sorghum grains contain a group of bioactive compounds beneficial to human well-being (Dykes, 2019; Kumari et al., 2021). Therefore, breeding for grain sorghum varieties as human nourishment has become a trending research topic among sorghum breeders in China. Developing broadly adapted sorghum varieties that have high and stable overall yields is the ultimate breeding goal of the breeders. However, yields are a complex trait and vary with genotypes (G) and environments (E), and even are challenged by the genotype × environment interaction (GEI) (Pour-Aboughadareh et al., 2022). The occurrence of significant GEI effects indicates differential expressions of phenotypes of genotypes across environments (da Silva et al., 2021). Yahaya et al. (2023) identified significant GEI effects (*p*< 0.05) on sorghum genotypes in variable environments. Similar reports have been presented that in the presence of significant GEI (*p*< 0.05), newly developed grain sorghum and sweet sorghum lines’ yields were subjected to the influences of the GEI (*p*< 0.05) across a range of environments (de Souza et al., 2021; Wanga et al., 2022). Consistently, the present study reported large variations for grain yields and yield related traits (**Table 3**), and revealed notably GEI effects (*p*< 0.001) on these traits in the METs of 2020 and 2021 (**Table 4**). ANOVA of the current study detected the same fact as reported by Farias et al. (2016) that significant E effects accounted for a proportion of the total effects much higher than both G and GEI effects for all the traits. It suggested that differences in phenotypic expressions of the traits were predominantly caused by environmental factors while genotypes did have impacts on phenotypic differences but in less magnitudes than environmental factors. Additionally, it was inferred from the low GEI proportion in the total variance that yield ranks of the sorghum genotypes showed limited fluctuation in all test locations. Nevertheless, Rono et al. (2016) and Kumar et al. (2014) pointed out that the presence of significant GEI effects on yields caused difficulties screening for superior lines by reducing screening efficiency. Thus, significant GEI effects need to be partitioned and further reconnoitered.

### 4.2 AMMI model for identification of superior genotypes

AMMI model is one of the powerful statistical tools that have been widely utilized for the analysis of GEI effects (Rodrigues et al., 2016). By the application of AMMI model in the present study, the significant GEI effects were partitioned into 12 multiplicative terms (IPCAs), the first two of which had significant effects (**Table 5**), suggesting that the interaction between the 13 grain sorghum genotypes and the seven locations in two consecutive growing seasons was predicted by the IPCA1 and the IPCA2 which accounted for 66.3% of the total variation. Our findings were consistent with the results of studies by Liu et al. (2022) and Khan et al. (2021). The two significant IPCAs detected in the present study are sufficient for the identification of superior genotypes, because Gauch and Zobel (1996) validated the sufficient accuracy of projecting the AMMI model with first two significant IPCAs. Significant IPCAs laid the foundation to carry out the AMMI for further stability analysis (Gauch, 2013). To assess the yield stability of the sorghum genotypes, IPCA scores and AMMI stability estimates were used in the present study. According to Habtegebriel (2022), IPCA1 score of each sorghum variety could be used as a parameter to judge the yield stability of the sorghum varieties. Mushoriwa et al. (2022) and Mwiinga et al. (2020) thought that genotypes with IPCA1 scores close to zero were regarded as stable genotypes. In view of that, the yield stability of the sorghum varieties was arranged in the order of G13 > G3 > G9 > G10 > G2 > G4 > G8 > G12 > G1 > G6 > G5 > G7 > G11. To confirm the order, ASV and WAASBY for each sorghum genotype was computed. According to the stability indexes together with the IPCA1 scores, G3, G9, G10, and G2 had small ASVs and therefore were classified as the well adapted varieties, whereas G6, G5, G7, and G11 were found to be unstable varieties.

The AMMI model generates AMMI1 and AMMI2 biplots for evaluating the significant multiplicative interactions of agronomic traits between genotypes and environments in multi-environment field trials. AMMI1 biplot gives visual information on yield potential and stability performance of genotypes under evaluation (Esan et al., 2023). In the AMMI1 biplot the ordinate and abscissa axes stand for the influence of environment effects and the mean yields, respectively (Olivoto et al., 2019). Esan et al. (2023) and Habtegebriel (2022) reported that genotypes located on the right side of the origin of the biplot along the abscissa axe had greater yields whereas those on the left side had lower yields, and that the influences of environments are larger when genotypes are arranged farther from the abscissa axe. Their results agree with our findings in the present study that G3, G10, G11, and G1 located to the right side of the origin had higher mean yields, G13, G3, G10, G2, and G9 relatively close to the abscissa had higher stability, among which G3 and G10 were thought as superior ones of both high yields and stability (**Figure 2**).

Unlike the AMMI1 biplot, AMMI2 biplot projects the interrelationships of genotypes and environments by using IPCA1 and IPCA2 scores. As described by Hossain et al. (2023) and Ljubicic et al. (2023), in the AMMI2 biplot deviations from the origin of the biplot represents the magnitudes of GEI effects on genotype, to be more explicitly, the farther away from the origin the greater the influence of GEI effects and vice versa, The present study applied a similar evaluation approach reported by Habtegebriel (2022) and identified G3, G10, and G2 to be the widely adapted genotypes of high environmental stability for their yield performances while G1, G7, G5, G13, and G11 as the ones of weaker stability because of their farther distances from the origin (**Figure 3**).

### 4.3 GGE biplot analysis evaluating test environments and genotypes

GGE biplot analysis has been proved a robust and miscellaneous statistical tool for evaluation of test environments and genotypes as well as recommendation of genotypes to specific environments (W Yan and Kang, 2003). According to Yan and Tinker (2006), GGE biplot analysis uses principal component analysis (PCA) to decompose the differential responses of genotypes under multi-environments and project the genotypic main effects (PC1) and the GEI effects (PC2) onto the abscissa and the ordinate axes of the biplot, respectively. In this way, evaluation of genotypes’ performances as well as mega environments identification can be visually and straightforward achieved (Zhang et al., 2016). Akcura et al. (2011) reported that the threshold of variations explained by the first two PCs derived from the PCA for a more trustworthy biplot interpretation should be more than 50% of the total variations. Comparable findings were recorded in the present study that PC1 and PC2 jointly captured 71.39% of the total variation for yields (**Figure 4**), much higher than the reported threshold for GGE biplot analysis, demonstrating superior fitness of our MET data and the analytical model as well as high reliability of the explanation of genotypes by the biplot analysis.

As a versatile investigative tool, GGE biplot analysis provides insights into the interrelations of test environments and discriminating ability to effectively distinguish phenotypic expressions of genotypes’. As described by Yan and Tinker (2006), relations between test environments can be inferred by comparing the included angles of two intersected environmental vectors in the biplot. In the study of soybeans by Habtegebriel (2022), environmental vectors that had included angles less than 90 degrees had positive correlations suggesting the similar genotype ranking under these environments which could be ascribed to similar environmental factors under which field trials were carried out. Smaller angles between environmental vectors meant more comparable genotypic ranking and therefore environmental conditions. When the included angles of environmental vectors were larger than 90 degrees, environments became negatively correlated indicating the divergence of genotype ranking that might be caused by dissimilar climatic conditions. Followed by the same evaluation criterion, positive correlations were found for five groups of environments (**Figure 4**), indicating that genotypes under the same group of environments could have similar genotypic rankings based on mean yield performances. Furthermore, irrelevant and contrary associations between test environment groups were revealed for the Group III and the Group V, and the Group IV (**Figure 5**). These types of environments could complicated the selection of ideal genotypes and push up costs for field tests and therefore Zhang et al. (2016) suggested removing such environments from test environments. Also notably, E7 and E14 were the test environments that were distinct from other test environments as revealed by the included angles between them. What is more, the two environments are at the same location of Yili in two different years, a place that is much unlike other test locations, characterized by distinct meteorological conditions of large differences between day and night temperatures, long sunshine hours, and low humidity, and suitable for high yielding potential development of crops.

Another feature of the GGE biplot model with respect to environmental evaluation is the ability to estimate the discriminating power plus the representation of average environments for test environments (Yan and Tinker, 2006; Oladosu et al., 2017). Esan et al. (2023) reported the use of GGE biplot analysis model to recognize test environments with higher capacity to discriminate among test genotypes with test environments that were more representative of the test environments. As stated in the earlier reports, longer environmental vectors in the biplot stood for greater ability to clearly distinguish between genotypes, on the other hand, wider divergences of environmental vectors from the AEC, revealed less representativeness of test environments. In the present study, E8 was the only one environment that had the most insubstantial ability to discriminate between genotypes, whereas E7 had the most exceptional competence of differentiate genotypes because it had the longest environmental vector (**Figure 5**). Environments such as E1, E2, E3, E4, E9, E10, and E11 were classified as a group with good discriminativeness while environments of E5, E6, E12, E13, and 14 had excellent discriminativeness. For environmental representativeness, E1, E3, E4, E5, E6, and E12 had very little deviations from the AEC, indicating their high levels of representativeness of environments. It is believed that Ideal test environments should have the extraordinary ability to distinguish each genotypes by fully presenting the differential phenotypic expression of a certain trait and have good representation of all test environments (W.K. Yan et al., 2000). In this sense, E5 (Yulin 2020), E6 (Pingliang 2020), and E12 (Yulin 2021) are outstanding test environments and furthermore Yulin is a location suitable for selecting superior genotypes. Test environments worthy of noting also include E7 (Yili 2020) and E14 (Yili 2021) which are at the same location of Yili. Although the two environments were relatively far from the AEC representing minor dissimilarity in environmental characteristics, it had stronger forces to distinguish phenotypic expressions of the sorghum genotypes.

Another core functionality of the GGE biplot model is to evaluate phenotypic express of genotypes over a series of environments and identify high yielding stable genotypes. Those exceptional genotypes are reflected in the “Mean vs. Stability” view of the GGE biplot, and with the “Ranking Genotypes” view desirable genotypes can be selected (Esan et al., 2023). In the Mean vs. Stability” view, the AEC starts from the left side of the biplot and points to the right direction of larger means for a trait suggesting that genotypes located on the right have larger means and thus better performance. On the other hand, vertical distances of genotypes onto the AEC indicate how much the GEI effects are imposed on the genotypes (Khazaei et al., 2022). In other words, the farther away from the AEC, the more unstable of a genotype. Accordingly, our present study revealed G13, G3, and G10 as the stable genotypes because of relatively shorter distances from the AEC, while, in terms of mean performances of yields, G3 and G10 excelled all other genotypes placed far on the left of the AEC (**Figure 6A**). The “Ranking Genotypes” view (**Figure 6B**) offers an alternative perspective to better understand the genotypic rankings based on yield means. The center of the concentric circles is positioned on the AEC. Genotypes situated on the concentric circles closer to the center have upper ranks and genotypes on the same concentric circle have same rank. With that stated, G3 and G10 are the two genotypes that have the first and second ranks in terms of yield means (**Figure 6B**). de Figueiredo et al. (2015) and Rodrigues et al. (2022) successfully selected ideal genotypes of soybean cultivars and maize cultivars, respectively, by implementing the selecting procedures of the GGE biplot analysis.

The polygon view, namely, “Which-Won-Where” view, of the GGE biplot delivers evidence for the recommendation of genotypes for specific environments (Solonechnyi et al., 2015). According to Rakshit et al. (2014), the biplot is divided into several quadrants by the dotted lines. Test environments enclosed in the same quadrant form the same group of environments. Vertexes of the polygon located in each quadrant or environment group represent the first rank of genotypes in the environment groups. As shown in **Figure 7** of the present study, four quadrants were observed with only two groups of environments identified. E7 and E14 were classified into one group of environments with other environments into the other group. The classification of E7 and E14 into the same group of environments agreed with the similar characteristics of the two environments as revealed in **Figures 4**, **5**. It could be inferred that environments at the location of Yili have unique distinctions that might be caused by the meteorological factors due to the distinctive geographical location of Yili. Additionally, in the polygon view, G11 was at the vertex in the quadrant formed by E7 and E14, demonstrating that G11 had the best yield performance, was a genotype well adapted to the environments at Yili, and could be promoted to Yili. On the other hand, G3 was the exceptional genotype that had the highest yields and the most extensive adaptability, and it could grow well across a wide range of environments. These findings supplemented the result of G3 and G10 being the superior cultivars by AMMI analysis and GGE biplot analysis for the mega-environment delineation.

### 4.4 Comparison of AMMI and GGE biplot analyses

AMMI model and GGE biplot model are two popular statistical algorithms coping with MET data and assisting in the better understanding of GEI effects on phenotype expressions of agronomic traits. Both models use the singular value decomposition (SVD) as the essence to break down and scrutinize the GEI effects (Solonechnyi et al., 2015). The present study first employed AMMI model to study the MET data. With the AMMI model, significant GEI was partitioned into two significant IPCAs to produce AMMI1 and AMMI2 biplots for the evaluation of performance and stability of genotypes. Results showed that G3 and G10 were observed by the AMMI model as the ideal sorghum cultivars which was confirmed with the AMMI stability estimates such as ASV and WASSBY. To verify the findings by AMMI model, GGE biplot analysis was adopted to study the MET data and select superior sorghum cultivars since both models are under debate on the effectiveness (Yan et al., 2007; Gauch et al., 2008). In the present study, the GGE biplot model confirmed the results by AMMI model and exhibited sophisticated detecting powers over the AMMI biplots for identifying G3 and G10 as the superior sorghum cultivars. The “Mean vs. Stability” and “Ranking Genotypes” views of GGE biplot analysis revealed detailed information on the mean performance, stability, and even the ranking of G3 and G10 more efficiently and directly than the AMMI model (Esan et al., 2023). Additionally, the views of “Discriminativeness vs. Representativeness” and “Relationship among Environments” evaluated the test environments and identified distinct test environments and locations providing useful information for deploying such MET in the future. The polygon view of the GGE biplot analysis also classified the environment groups as discovered in the “Discriminativeness vs. Representativeness” and “Relationship among Environments” views, and identified superior lines specifically adapted to certain environments, for example, G11 for the location of Yili while G3 for all the other locations. Most of the findings revealed by the GGE biplot analysis were consistent with the those by the AMMI model, however, GGE biplot gained advantages over the AMMI model for identifying high yielding and stable genotypes, and evaluating genotypes adaptability in each environment (Esan et al., 2023).

### 4.5 Trait correlations and agronomic features of the identified superior grain sorghum varieties

Associations between yield and agronomic traits have been broadly documented. For example, Andiku et al. (2022) and Sulistyo et al. (2018) presented significant positive correlations between yield, plant height, panicle length, and hundred grain weight in sorghum and soybeans, respectively. Similarly, it has been recorded that maize yield was notably positively correlated with some agronomic traits such as plant height, grain weight (Ren et al., 2022). Positive correlations between target traits suggest the simultaneous increase or decrease of these traits (Sulistyo et al., 2018). However, when target traits exhibit significant negative associations, compromise should be made for selecting one of those traits (Wang et al., 2017). In our own case, YLD showed significant negative correlations with PL and TSW and even trivial correlations with DTM and PH (**Figure 1**). The result demonstrated larger yield with shorter panicle length and lower grain weight on the contrary to generally reported correlations between yield and other agronomic traits. It could be attributable to the limited sample size under evaluation or the test varieties’ own characteristics such as wider but shorter panicles and larger number but less weight of grains per panicle in the present study. Also, the reason could be that the general breeding purpose of grain sorghum in China has turned to reshaping efficient sorghum plant architecture, i.e., overall smaller plant size especially shorter plant height with higher yield (Li et al., 2021). For that purpose, shorter sorghum varieties with larger yields are preferred and have been improved, for example, those test varieties in the present study.

As the superior grain sorghum variety adapted to all environments and selected in the present study, G3 (Liaoza No.53) has the largest mean yield of 9.33 ± 2.68 t·ha^-1^, a higher level of thousand seed weight is 29.99 ± 4.45 g, the second lowest plant height of 150.94 ± 16.87 cm, and the second shortest panicle length 29.52 ± 2.68 cm. This sorghum variety needs 127.36 ± 18.51 d to reach full maturity. Whereas, G10 (Jinza 110), the second promising grain sorghum variety, has the second largest unit area production of 9.24 ± 2.88 t·ha^-1^, the least thousand seed weight of 27.10 ± 4.73 g, low-ranged plant height of 153.57 ± 25.54 cm, and below-averaged panicle length of 30.01 ± 3.12 cm. It can be harvested in 126 ± 16.47 d after sprouting. Unlike G3 and G10, G11 is much weaker than G3 and G10 and well-grown solely in a certain environment. This variety has a lower mean yield of 8.66 ± 3.90 t·ha^-1^. Coefficient of variation for G11’s yield is up to 45.10% indicating less stable field performance across all environments than G3 (28.66%) and G10 (31.21%). Its thousand seed weight is 28.40 ± 4.89 g lower than that of G3 and G10. However, G11 has a taller plant height of 175.44 ± 24.50 cm and longer panicle length of 32.31 ± 5.31 cm. The variety has a growth duration of 128.07 ± 19.47 d for maturity. All grain sorghum varieties have high yields and short plant heights within the range of 120 cm to 180 cm which is believed to be the best plant height for machinery harvest (Li et al., 2021). Additionally, their duration of growth is around 120 days suitable for growing in the spring sowing and late maturing region for sorghum production of North China while G11 is strongly recommended for local production at Yili within the region mentioned above.

## 5 Conclusion

The 13 grain sorghum cultivars under current investigation exhibited significant variations in response to the 14 test environments at the seven distinct geographical locations. GEI effects were the cause of the differential expressions of YLD, and yield related traits such as DTM, PL, PH, and TSW. G3 and G10 were the selected superior grain sorghum cultivars with high yields and high stability. G3 was a high yielding and widely adapted grain sorghum cultivars. It outperformed all other cultivars in both yield and stability and was recommended to grow at Shenyang, Chaoyang, Jinzhou, Jinzhong, Yulin, and Pingliang while G11 had the best performance than any other varieties and was the one specifically adapted to the environments at Yili and therefore G11 could be promoted to the region of Yili.

## Data availability statement

The original contributions presented in the study are included in the article/**Supplementary Material**. Further inquiries can be directed to the corresponding author.

## Author contributions

RW: Conceptualization, Investigation, Methodology, Writing – original draft, Formal Analysis, Visualization, Writing – review & editing. HW: Investigation, Data curation, Formal Analysis, Writing – review & editing. SH: Writing – original draft, Writing – review & editing. YZ: Data curation, Formal Analysis, Investigation, Writing – review & editing. EC: Data curation, Investigation, Writing – review & editing. FL: Data curation, Formal Analysis, Investigation, Writing – review & editing. LQ: Writing – review & editing. YY: Writing – review & editing. Y’aG: Writing – review & editing. BL: Data curation, Investigation, Writing – review & editing. HZ: Conceptualization, Investigation, Methodology, Supervision, Writing – original draft, Writing – review & editing.
