# Supplementary material for: Assessment of yield performances for grain sorghum varieties by AMMI and GGE biplot analyses
Source: Front Plant Sci. 2023 Oct 30;14:1261323. doi: 10.3389/fpls.2023.1261323 (PMC10642804; doi:10.3389/fpls.2023.1261323)
Supplement: Supplementary file 3 [file Table_3.docx]

Please set your own working directory

setwd('xxxx')

#Load data

dat <- read.table('correlation.txt', header = T, sep = '\t')

#Load the 'metan' package into R console, install it prior to loading

library(metan)

#Use the code below to generate the correlogram plot

corr_plot(dat, upper = 'corr', lower = 'scatter', diag = T, diag.type = 'histogram',

size.point = 0.5, maxsize = 4, minsize = 4, smooth = T, size.line = 0.5, digits = 3, export = T, file.type = 'tiff',

file.name = 'correlation', resolution = 300)
