# Supplementary material for: Assessment of yield performances for grain sorghum varieties by AMMI and GGE biplot analyses
Source: Front Plant Sci. 2023 Oct 30;14:1261323. doi: 10.3389/fpls.2023.1261323 (PMC10642804; doi:10.3389/fpls.2023.1261323)
Supplement: Supplementary file 4 [file Table_4.docx]

# Set your own working directory where the raw data file (stored in an excel file, convert it to text file # before use) is located

setwd('xxxx')

# Load data

dat <- read.table('raw_data.txt', header = T, sep = '\t')

# Load metan package

library(metan)

# Ran AMMI analysis

model <- performs_ammi(dat, env = env, rep = rep, gen = var, resp = Yield)

model

model_indexes <- ammi_indexes(model)

model_indexes

print.table(model_indexes$Yield)

# Generate AMMI1 biplot

plot_scores(model, size.tex.env = 5, size.tex.gen = 5, size.tex.lab = 15, size.shape.env = 4, size.shape.gen = 4, leg.lab = c("Env", "Var"), size.segm.line = 0.5)

# Generate AMMI2 biplot

plot_scores(model, type = 2, polygon = F, size.tex.env = 5, size.tex.gen = 5, size.tex.lab = 15, size.shape.env = 4, size.shape.gen = 4, leg.lab = c("Env", "Var"), size.segm.line = 0.5)
