# Supplementary material for: Assessment of yield performances for grain sorghum varieties by AMMI and GGE biplot analyses
Source: Front Plant Sci. 2023 Oct 30;14:1261323. doi: 10.3389/fpls.2023.1261323 (PMC10642804; doi:10.3389/fpls.2023.1261323)
Supplement: Supplementary file 5 [file Table_5.docx]

# Set your own working directory where the raw data file (stored in an excel file, convert it to text file # before use) is located

setwd('xxxx')

# Load data

dat <- read.table('raw_data.txt', header = T, sep = '\t')

# Load metan package

library(metan)

# Run GGE biplot model with singular value partitioned to genotype

model1 <- gge(dat, env = env, gen = var, resp = Yield, svp = 'genotype')

# Generate which-won-where view

www <- plot(model1, type = 3, size.shape.win = 6, size.text.win = 6, size.line = 0.5,size.text.env = 5, size.text.gen = 5, size.shape = 4, col.env = 'red', col.gen = 'blue', leg.lab = c('Env', 'Var'), size.text.lab = 16)

www

# Generate mean vs. stability view

meanstability <- plot(model1, type = 2, col.gen = 'blue', col.env = 'red', size.text.env = 5, size.text.gen = 5, size.line = 0.5, leg.lab = c('Env', 'Var'), size.text.lab = 16)

meanstability

# Generate ranking genotypes view

rankgeno <- plot(model1, type = 8, col.env = 'red', size.shape = 4, size.text.env = 5, size.text.gen = 5, size.line = 0.5, leg.lab = c('Env', 'Var'), size.text.lab = 16)

rankgeno

# Run GGE biplot model with singular value partitioned to environment

model2 <- gge(dat, env = env, gen = var, Yield)

# Generate discriminativeness and representativeness view

disvsrep <- plot(model2, type = 4, col.gen = 'blue', col.env = 'red', size.text.env = 5, size.text.gen = 5, size.shape = 4, size.line = 0.5, leg.lab = c('Env', 'Var'), size.text.lab = 16)

disvsrep

# Generate relationship among environments view

rlamenv <- plot(model2, type = 10, col.env = 'red', size.text.env = 5, size.text.gen = 5, size.shape = 4, size.line = .5, leg.lab = c('Env', 'Var'), size.text.lab = 16)

rlamenv
